# The exploration of interoception construct in COVID-19 survivors

**DOI:** 10.1192/j.eurpsy.2023.1673

**Published:** 2023-07-19

**Authors:** G. D’Orsi, M. Palladini, A. Scalabrini, M. G. Mazza, S. Poletti, P. Rovere-Querini, F. Benedetti

**Affiliations:** 1 Psychiatry and Psychobiology Unit, Division of Neuroscience, IRCCS San Raffaele Hospital; 2 Psychiatry and Psychobiology Unit, Division of Neuroscience, IRCSS San Raffaele Hospital; 3 Vita-Salute San Raffaele University, Milan; 4 Department of Human and Social Sciences, University of Bergamo, Bergamo; 5 Immunology, Transplantation and Infectious Diseases, IRCSS San Raffaele Hospital, Milan, Italy

## Abstract

**Introduction:**

The new coronavirus disease (COVID-19) has important physical and mental health implications at short and long term. Some inflammatory parameters are implicated in the maintenance of psychiatric symptoms, especially those of anxiety and depression. Additionally, growing literature attributes a role to interoception in several mental health conditions.

**Objectives:**

We investigated the involvement of the interoception in COVID-19 survivors and its possible associations with psychopathological and inflammatory variables.

**Methods:**

Our study included 57 people surviving COVID-19 at one month follow-up after recovery. Individual interoceptive accuracy (IA) measure was obtained through heart-beat perception task. A measure of accuracy in external time perception (TA) was also obtained asking people to mentally produce a duration of 10s. Each participant completed State-Trait Anxiety Inventory - STAI-Y; Zung Self-Rating Depression Scale - ZSDS; Beck Depression Inventory - BDI-II; Impact of Events Scale - IES-R and Multidimensional Assessment of Interoceptive Awareness - MAIA. Peripheral inflammation markers were obtained in a subsample of 40 people by a blood sampling conducted at the time of admission and discharge from hospital. Correlation, regression and GLM analyses were performed with SPSS. Mediation analysis were performed with Hayes’ Process tool.

**Results:**

TA is not associated with IA, symptomatological measures and bodily awareness. Trusting is the only aspect of body awareness associated with IA (p=.021). Noticing (p=.010), Not-distracting (p=.009), Not-worrying (p=.012) and Trusting (p=.001) predict anxiety psychopathology. Poor IA predict anxiety symptomatology (p=.004) and part of this effect is mediated by Trusting [Fig.1]. In the end, platelets count at the time of hospitalization negatively correlates with anxiety symptoms (p=.003).

**Image:**

**Figure:**
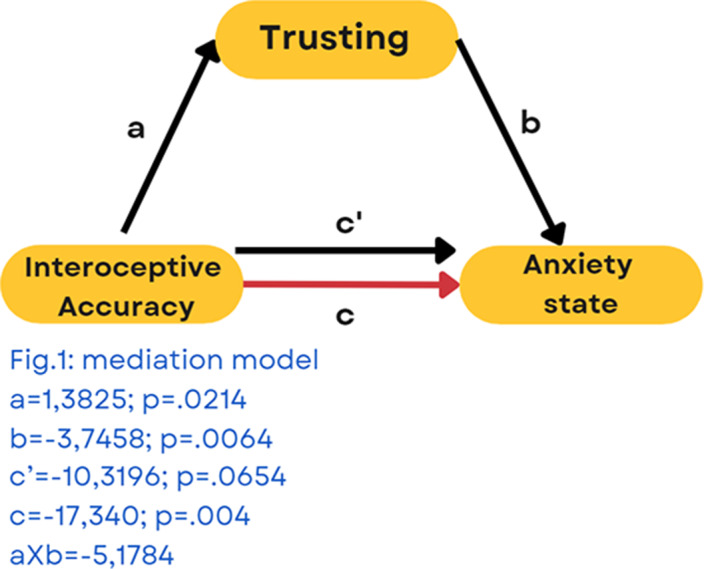

**Conclusions:**

COVID-19 hospitalization could be considered a psychophysical traumatic experience which involved mental and physical health and the connection and integration between them. It’s necessary to deepen the different facets of body awareness and IA in post-covid stages and to study how interoceptive dimensions change over time. Further research is needed to investigate the specific role of platelets in prominent anxiety psychopathology detected in COVID-19 survivors, wondering about their possible involvement in the dysfunctional interoception process too.

**Disclosure of Interest:**

None Declared

